# Development of a computerized adaptive test to assess entrepreneurial personality

**DOI:** 10.1186/s41155-020-00144-x

**Published:** 2020-05-11

**Authors:** Álvaro Postigo, Marcelino Cuesta, Ignacio Pedrosa, José Muñiz, Eduardo García-Cueto

**Affiliations:** 1 Department of Psychology, University of Oviedo, Plaza Feijoo s/n, 33003 Oviedo, Spain; 2 Parque Científico Tecnológico (CTIC), Gijón, Spain

**Keywords:** Entrepreneurial personality, Evaluation, Computerized adaptive test, Adults

## Abstract

**Background/objective:**

Entrepreneurial behavior is of great importance nowadays owing to its significance in the generation of economic, social, personal, and cultural wellbeing. This behavior is influenced by cognitive and personality characteristics, as well as by socioeconomic and contextual factors. Entrepreneurial personality is made up of a set of psychological traits including self-efficacy, autonomy, innovation, internal locus of control, achievement motivation, optimism, stress tolerance, and risk-taking. The aim of this research is the development of a computerized adaptive test (CAT) to evaluate entrepreneurial personality.

**Method:**

A bank of 120 items was created evaluating various aspects of the entrepreneurial personality. The items were calibrated with the Samejima Graded Response Model using a sample of 1170 participants (*M*_age_ = 42.34; SD_age_ = 12.96).

**Results:**

The bank of items had an essentially unidimensional fit to the model. The CAT exhibited high accuracy for evaluating a wide range of *θ* scores, using a mean of 16 items with a very low standard error (*M* = 0.157). Relative validity evidence for the CAT was obtained with two additional tests of entrepreneurial personality (the *Battery for the Assessment of the Enterprising Personality* and the *Measure of Enterpreneurial Tendencies and Abilities*), with correlations of .908 and .657, respectively.

**Conclusions:**

The CAT developed has appropriate psychometric properties for the evaluation of entrepreneurial people.

Entrepreneurship has been on the rise in recent years in developing countries and has become consolidated in mature economies owing to its importance in the modern economy (GEM, 2018, 2019). Organizations such as the Global Entrepreneurship Research Association monitor entrepreneurship annually to analyze its social and economic impact (GEM, 2019).

The study of entrepreneurship has attracted research attention and in recent years has become consolidated as a multidisciplinary field bringing together three main perspectives: economics (Obschonka et al., 2015), sociology (Chell, 2008), and psychology (Chandra, 2018; Gorgievski & Stephan, 2016). All kinds of individual variables have been examined from the psychological perspective, especially personality characteristics (Omorede, Thorgren, & Wincent, 2015), such as self-efficacy (Newman, Obschonka, Schwarz, Cohen, & Nielsen, 2019), locus of control (Asante & Affum-Osei, 2019), and optimism (Adomako, Danso, Uddin, & Damoah, 2016), among others. There are two overarching strategies that mark research into entrepreneurial personality: on the one hand are researchers focusing on general, Big Five-type personality traits (Brandstätter, 2011; Zhao, Seibert, & Lumpkin, 2010), and on the other hand are those who concern themselves with more specific traits related to entrepreneurial personality (Muñiz, Suárez-Álvarez, Pedrosa, Fonseca-Pedrero, & García-Cueto, 2014), based on the model of Rauch and Frese (2007). Researchers in this latter group argue that specific traits provide better predictive capability than the general traits (George, Parida, Lahti, & Wincent, 2016; Leutner, Ahmetoglu, Akhtar, & Chamorro-Premuzic, 2014). Once specific traits were identified (e.g., self-efficacy, internal locus of control, achievement motivation, risk taking), a variety of instruments have been proposed to evaluate entrepreneurial personality (Suárez-Álvarez & Pedrosa, 2016). Standouts include the *Entrepreneurial Mindset Profile* (EMP; Davis, Hall, & Mayer, 2016), the *High Entrepreneurship, Leadership and Professionalism Questionnaire* (HELP; Di, Di, Bucci, & Gori, 2016), the *Measure of Enterpreneurial Tendencies and Abilities* (META*;* Almeida, Ahmetoglu, & Chamorro-Premuzic, 2014), and the *Battery for the Assessment of the Enterprising Personality* (BEPE) for young people (Muñiz et al., 2014; Suárez-Álvarez, Pedrosa, García-Cueto, & Muñiz, 2014) and adults (Cuesta, Suárez-Álvarez, Lozano, García-Cueto, & Muñiz, 2018). These measuring instruments all have their strengths and weaknesses. They can all evaluate different specific traits of entrepreneurial personality in a single instrument, are reliable, and have sufficient evidence of validity (see, Suárez-Álvarez & Pedrosa, 2016). On the other hand, most of them do not use the latest psychometric developments, such as Item Response Theory (IRT) models, and thus suffer from the drawbacks associated with that (Van der Linden, 2016), such as the lack of invariance with respect to instruments extracted from the same bank of items, as well as the sample used to estimate the properties of the test. IRT also offers a more rigorous methodological framework which allows computerized adaptive testing (CAT) to be used.

The fundamental thing about CAT is that it allows tests to be adapted to the person being evaluated, which has clear advantages, significantly reducing testing time without losing accuracy (Abad, Olea, Ponsoda, & García, 2011; Muñiz, 2018; Olea, Ponsoda, & Prieto, 1999; Van der Linden & Glas, 2010), meaning rapid and accurate evaluations. A CAT allows items to be selected based on the participant’s responses to previous items, modifying the test to the test taker (De Ayala, 2009; Meijer & Nering, 1999). Due to these advantages, CAT testing has taken off exponentially in the last few decades (Zenisky & Luecht, 2016), particularly in a broad range of evaluation areas such as entrepreneurial personality in young people (Pedrosa, Suárez-Álvarez, García-Cueto, & Muñiz, 2016), personality from the Big Five model (Nieto et al., 2017), organizational climate (Menéndez, Peña-Suárez, Fonseca-Pedrero, & Muñiz, 2017), schizotypal personality (Moore, Calkins, Reise, Gur, & Gur, 2018), schitzotipy (Fonseca-Pedrero, Menéndez, Paino, Lemos-Giráldez, & Muñiz, 2013), and general intelligence (Herranz-Torres, 2017).

Despite the psychometric advantages of CAT, to date, nothing has been developed to evaluate entrepreneurial personality in adults. The objective of this study, therefore, is to develop a CAT for the evaluation of entrepreneurial personality for adults. The computerized adaptive test of entrepreneurial personality will provide clear psychometric and economic advantages over the classic forms of testing and will be an appealing and beneficial alternative in organizational environments, especially in a recruitment context, where its intended use would be to evaluate a large number of candidates in a very short time.

## Method

### Participants

The initial sample comprised 1324 participants recruited through a snowball procedure. The final sample was 1170 people, owing to low scores (less than 8 out of 10) in a scale controlling response quality, described in the “Instruments” section. The mean age of the sample was 42.34 years old with a standard deviation of 12.96, the minimum age was 18, and the maximum was 80. Over half (59.9%) were women. A minority (13%) were self-employed. Self-employed people were those who had set up a business and were working in it; non-self-employed people were those in a salaried position in either public or private entities.

### Instruments

#### Pool of items (BEPE-CAT)

The development process of the CAT used the following process (Muñiz & Fonseca-Pedrero, 2019). A team of eight experts in Psychometrics constructed an initial bank of 161 items, in Spanish, designed to measure the eight specific facets defining entrepreneurial personality: self-efficacy, autonomy, innovation, internal locus of control, achievement motivation, optimism, stress tolerance, and risk-taking (Cuesta et al., 2018; Rauch & Frese, 2007). For an item to be selected, all members of the expert group had to agree on its content. The items were in a Likert-type format with five response categories ranging from “completely disagree” to “completely agree.” All of the items were in a positive direction to minimize response bias (Suárez-Álvarez et al., 2018). How well the 161 items represented the content was evaluated by 15 experts in psychological evaluation (none of whom had been on the first development team) using a scale of 1 to 10 to indicate their level of agreement with the definition they were provided of the variable to measure. This team of 15 was made up of professors from the area of personality and psychological evaluation from various Spanish universities. Items with an average score below 8 were reformulated. Following that, 142 psychologists, selected through convenience sampling, with the sole criterion that they were graduates in Psychology, rated the suitability of each item on a scale of 1 to 10. Any item scoring below 9 was reviewed and revised. Once the 161 items were reviewed, a pilot study was performed with a sample of 132 participants, selected through convenience sampling, in order to perform a first study of how the items functioned. An exploratory factor analysis of each subscale was performed, using the polychoric correlation matrix and the method of generalized least squares. The items with factorial loadings below 0.30 and/or with discrimination indexes below .20 were eliminated iteratively one by one (Muñiz, Fidalgo, García-Cueto, Martínez, & Moreno, 2005). The final bank was made up of 120 items. Examples of the item bank are “I can make risky decisions” and “I like to face new challenges.”

#### Battery for the Assessment of the Enterprising Personality (BEPE)

The BEPE (Cuesta et al., 2018) is a battery made up of 80 items and measures eight dimensions (10 items per dimension): self-efficacy, autonomy, innovation, internal locus of control, achievement motivation, optimism, stress tolerance, and risk-taking. These eight dimensions combine to a common factor of entrepreneurial personality. This instrument has been developed from the pool of items presented in this study. According to Cuesta et al. (2018), the scale has the following reliability data: self-efficacy (*α* = .88), autonomy (*α* = .81), innovation (*α* = .88), internal locus of control (*α* = .85), achievement motivation (*α* = .86), optimism (*α* = .89), stress tolerance (*α* = .84), risk-taking (*α* = .87), and enterprising personality (*α* = .97), which are excellent according to CET-R (Hernández, Ponsoda, Muñiz, Prieto, & Elosua, 2016).

#### Measure of Entrepreneurial Tendencies and Abilities (META)

The META test (Ahmetoglu & Chamorro-Premuzic, 2013) has 44 items which measure four personality traits relevant to entrepreneurial success: proactivity, creativity, opportunism, and vision. The items are in a Likert-type format with five response categories ranging from “completely disagree” to “completely agree.” The reliability (Cronbach alpha) for the four scales is as follows; proactivity, .84; creativity, .83; opportunism, .86; and vision, .76 (Ahmetoglu & Chamorro-Premuzic, 2013). The present study obtained the following values for the alpha coefficient: proactivity (.70), creativity (.81), opportunism (.86), and vision (.76).

#### NEO Five Factor Inventory (NEO-FFI)

The NEO-FFI test (Costa & McCrae, 1985) is an inventory made up of 60 Likert-type items with five response categories from “totally disagree” to “totally agree.” It is made up of five scales (12 items per scale) following the *Big Five* personality model: Neuroticism, Extraversion, Openness, Agreeableness, and Conscientiousness. The adaptation to Spanish was carried out by Cordero, Pamos, and Seisdedos (2008). The reliability data for the scales are as follows (Cronbach alpha): neuroticism, .90; extraversion, .84; openness, .82; agreeableness, .83; and conscientiousness, .88 (Cordero et al., 2008). The present study obtained the following values for the alpha coefficient: neuroticism (.90), extraversion (.84), openness (.82), agreeableness (.83), and conscientiousness (.88).

#### Control of Attention Scale

The aim of this scale is to detect participants who respond randomly or thoughtlessly to the items in any of the instruments used. It is made up of 10 obvious prompts such as “in this item, choose the option completely agree.” Participants should respond correctly to all items. Participants who responded incorrectly to two or more items were eliminated. This meant that 11.6% of the sample (154 participants) were eliminated from the study.

### Procedure

An online application, developed ad hoc*,* was used for the aforementioned 120-item bank along with the other instruments. Informed consent was obtained from the participants, who were recruited via snowball sampling. Potential participants who met the inclusion criteria (workers over 18 years old) were personally contacted. They were asked to answer the online questionnaire and provide contact details for other potential participants. These new potential participants were asked to collaborate both in answering the questionnaire and in obtaining contact details for more new participants. The response process was open for 3 months (February to April, 2017). The average response time estimated in the test phase was 40 min. Participants did not receive any kind of reward for participating in the study. The anonymity of each participant in this study was scrupulously respected, confidentiality was maintained, and the ethical code of the Officials Colleges of Psychologists was followed.

### Data analyses

The unidimensionality of the responses to the item bank was checked via confirmatory factor analysis with cross-validation. The participants were divided into two similarly sized, random subsamples. A confirmatory factor analysis was performed with the first subsample (*n*_1_ = 589). With the second subsample (*n*_2_ = 581), the confirmatory analysis was repeated to demonstrate the convergence of the indices of fit obtained (Byrne, 2001; Jackson, Gillaspy, & Purc-Stephenson, 2009). We used Robust Maximum Likelihood as the estimation method (Kline, 2011). The indices of fit we used were *X*^2^/df, *Comparative Fit Index* (CFI), and *Root Mean Square Error of Approximation* (RMSEA), with fit being adequate when *X*^2^/df < 3, CFI > .90 y RMSEA< .08 (Kline, 2011). It should be noted that CFI is sensitive to the number of items, and it is not recommended for studying the unidimensionality of an item bank (Calderón-Garrido, Navarro-González, Lorenzo-Seva, & Ferrando-Piera, 2019; Cook, Kallen, & Amtmann, 2009); therefore, RMSEA and *X*^2^/df were the most suitable for looking at the fit. However, the CFI was a commonly cited index in the literature. MPlus 8 software was used to perform the confirmatory factorial analysis (Muthén & Muthén, 2017).

Differential Item Functioning (DIF) by gender was analyzed via logistical regression (Gómez-Benito, Hidalgo, & Zumbo, 2013; Hidalgo, Gómez, & Padilla, 2005; Zumbo, 1999). To calculate DIF, the SPSS24 (IBM Corp., 2016) statistical package was used.

The bank of 120 items was applied to the 1170 participants, estimating the information function of the bank, the standard error of measurement, the skill level of the participants, and performing calibration of all items. This was done using the Samejima Graded Response Model (Samejima, 1969).

Following that, two complementary studies were performed using simulation procedures. A sample of 130,000 participants was simulated, divided into 13 subsamples depending on their true score (*θ* between −3 y + 3) with intervals of 0.5. This sample responded first to the complete bank of items, and via the aforementioned Samejima Graded Response Model (Samejima, 1969), the deviation of the estimated ability was calculated compared to the real ability of each simulated participant, i.e., the measurement error for each ability level using the complete bank of 120 items. Secondly, the same sample responded to the CAT. The algorithm used to apply the CAT was as follows: (1) use a minimum of 10 items, (2) select as a starting criterion one of the items with an *a* parameter over 3, and (3) the stop criteria are a maximum of 35 items presented, or the reduction in error is less than 5% compared to the previous estimation.

All estimations were performed via maximum likelihood procedures. Depending on each participant’s response, in each step, the level of *θ* (Meijer & Nering, 1999) is estimated, selecting an item from the bank with the maximum information function for the estimated level of θ. Following that, a new *θ* and standard error (SE) are calculated for each participant, and the process is repeated until one of the stop criteria are met.

Finally, the algorithm was used to simulate the application of the CAT to the 1170 participants based on their responses to the complete item bank. In addition, the correlation between the *θ* from the participants in the 120 items and their estimated (*θ*) score by the CAT was calculated. All IRT analyses and the simulation were performed using MAGP software (García-Pérez, 2018).

To produce predictive validity evidence for the CAT, the correlation was calculated between *θ* estimated by the CAT and the participants’ scores in the BEPE, META, and NEO-FFI tests.

Finally, also as evidence of validity, we calculated whether there were statistically significant differences in the *θ* estimated by the CAT between the group of self-employed workers and those who worked for others, using the Student *t* test for independent samples.

## Results

Table 1 shows that the indexes of fit for the two subsamples were very similar, demonstrating convergence of the indices of fit, and serving as evidence of cross-validation with respect to the proposed factor structure. In addition, the RMSEA and *X*^2^/df were sufficient to confirm the essential unidimensionality of the responses to the bank of items. Although the values of the CFI index are relatively low, this may be due to the high number of items that we are working with, something which reduces the value of this indicator (Cook et al., 2009). The other two values are, in any case, adequate, which would confirm the fit of the model (Mosewich, Hadd, Crocker, & Zumbo, 2013).

**Table 1:** Confirmatory factor analysis of the item bank

CHI-2/DF		CFI		RMSEA (CI)	
N1	N2	N1	N2	N1	N2
2.27	2.30	.69	.69	.046 [.046, − .050]	.048 [.048, − .050]

*Note: DF* degrees of freedom, *CFI* Comparative Fit Index, *RMSEA* Root Mean Square Error of Approximation, *CI* confidence interval

Of the 120 items in the bank, only item 98 exhibited uniformly statistically significant DIF in relation to gender. However, the effect size of the DIF for this item was low.

**Fig. 1:**
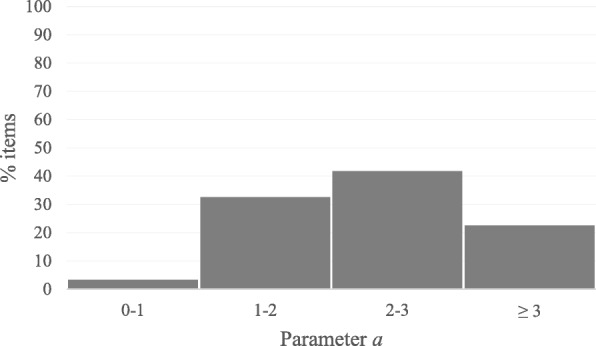
Distribution of the parameter *a* values of the items

With respect to the fit of the Samejima Graded Response Model, the analysis of the standardized residuals gave a mean of 0.14 and a standard deviation of 0.87 for the items as a whole. These results are close to the ideal values of a distribution of standardized residuals (*M* = 0; SD = 1). All the items were statistically significant (*p* < .05). Therefore, we can state that the bank fits the Samejima Graded Response Model (Samejima, 1969). The *a* parameter of the items exhibited appropriate values, between 0.66 and 4.59 (Fig. 1) (Baker, 1985). The *b* parameters for each item were adequate and scaled in the expected order, going from smaller to larger. The maximum information for each item was, in general, within a range of *θ* scores between − 1 and + 2.

**Fig. 2:**
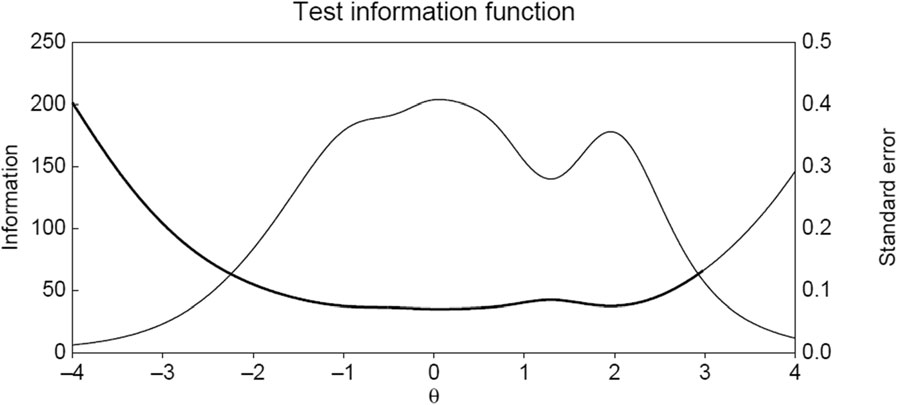
Test information function of the item bank

We used the standard error (SE) of estimated participants’ scores (*θ*) to evaluate the accuracy of the item bank. The information function was estimated for a range of scores between − 4 and + 4. This is shown in Fig. 2, where the thick line represents the error of estimation and the fine line represents the information provided by the item bank. The accuracy is sufficient, with a small error of estimation for all skill levels (*M* = 0.142), below 0.1 between scores of − 2 and + 2.5.

**Fig. 3:**
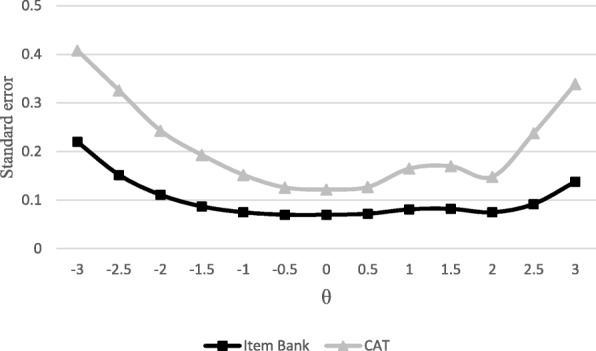
Comparison of standard error for the item bank and the CAT

Once the accuracy of the item bank had been demonstrated with empirical data, we examined how it functioned with larger, heterogeneous samples (130,000 participants). The simulation indicated that the item bank demonstrated better accuracy for *θ* levels between − 1.5 and + 2.5. Nonetheless, the errors of estimation were very small for all ranges of *θ* (*M* = 1.101). To confirm the functioning of the CAT, the same sample was simulated responding to the CAT. The result of the simulation was that the use of the items ranged between a mean of 12 for *θ* = 3 and 19 for *θ* = − 0.5. We found a mean error of estimation of 0.212, larger than when the complete item bank was applied (*M* = 0.101), but even so, it may be considered particularly small, bearing in mind the number of items used, suggesting a small loss of information. That may be seen in Fig. 3, which shows a comparison of the error of estimation applying the full item bank and applying the CAT, with large, heterogeneous samples.

Table 2 shows the percentage use of each of the CAT items. Items used as starting criteria due to their high discriminatory power *(a* ≥ 3) are highlighted.

**Table 2:** Frequency of computerized adaptive test item use

Items	Frequency (%)	Items	Frequency (%)	Items	Frequency (%)
Item 1	0	Item 41	2.8	Item 81	0.1
**Item 2**	49	Item 42	9.4	Item 82	15.5
**Item 3**	59.5	**Item 43**	51.8	**Item 83**	57.7
Item 4	30	**Item 44**	8.5	Item 84	0
**Item 5**	9.5	Item 45	0	Item 85	0
**Item 6**	27	Item 46	13.1	Item 86	0
Item 7	0.3	Item 47	0.6	Item 87	0
**Item 8**	15.6	**Item 48**	20.6	Item 88	0
**Item 9**	54.6	Item 49	0	**Item 89**	54.5
Item 10	0	Item 50	2.2	Item 90	0.1
Item 11	19.4	Item 51	0.5	**Item 91**	54.1
**Item 12**	12.2	Item 52	8	Item 92	0.8
**Item 13**	51.5	Item 53	5.2	Item 93	0
**Item 14**	58.3	Item 54	3.3	Item 94	0
**Item 15**	39.9	Item 55	1.82	Item 95	0
Item 16	4	Item 56	0.1	Item 96	1.7
Item 17	8.7	Item 57	23	Item 97	0.4
Item 18	0.7	Item 58	8.5	Item 98	3.6
Item 19	0	Item 59	0	Item 99	3.4
Item 20	3.6	Item 60	11.5	Item 100	0.3
Item 21	1.6	Item 61	14.7	Item 101	1.6
Item 22	2.1	**Item 62**	52.7	Item 102	15.2
Item 23	4.3	Item 63	19.4	Item 103	1.3
Item 24	23.3	Item 64	17.2	Item 104	0.3
Item 25	11.4	Item 65	0.7	Item 105	0
Item 26	23.8	**Item 66**	20.4	Item 106	6.1
Item 27	0.5	**Item 67**	47	**Item 107**	54.1
Item 28	15	Item 68	0	Item 108	7.7
Item 29	5.4	Item 69	6	Item 109	11.6
Item 30	28.4	Item 70	4.6	Item 110	0.9
Item 31	7.4	**Item 71**	54.1	Item 111	0.1
Item 32	1	**Item 72**	43.8	**Item 112**	26.1
**Item 33**	19.1	Item 73	11	Item 113	0.4
Item 34	0	Item 74	1.4	Item 114	20.3
Item 35	15.5	Item 75	0.3	Item 115	1.3
Item 36	0.	Item 76	0	Item 116	0.1
**Item 37**	20.5	Item 77	0	Item 117	9.4
Item 38	0	Item 78	0.1	**Item 118**	34.2
Item 39	2.2	Item 79	0	Item 119	0
Item 40	16.3	**Item 80**	4.9	Item 120	8.1

Items in bold are those used as starting criteria due to their high *a* parameters

We simulated applying the CAT to the 1170 participants, based on their responses to the full item bank. The CAT used between 10 and 26 items, with a mean of 16 items used, and used fewer than 20 items with 78.4% of participants. A mean error of estimation was produced (*M* = 0.157) which was very small given the low number of items used. The correlation between *θ* for the participants with the 120-item bank and their estimated (*θ*) score in the CAT was very high (*r*_*θ*1–*θ*2_ = .948).

For evidence of validity, we calculated the Pearson correlation between the CAT estimated *θ* scores of the 1170 participants and their scores in the BEPE, META, and NEO-FFI. The *θ* scores from the CAT correlated very strongly with the four META dimensions and with the META total, which demonstrates high convergence between the two instruments. In terms of general personality traits, the CAT *θ* scores showed strong correlations with conscientiousness, extraversion, and neuroticism, with the latter correlation being negative. Lastly, the CAT *θ* scores showed very strong correlations with the BEPE dimensions and the overall BEPE (Table 3)

**Table 3:** Pearson correlations between *θ* CAT scores and BEPE, META, and NEO-FFI tests

NEO-FFI	*θ* CAT	META	*θ* CAT	BEPE	*θ* CAT
Agreeableness	.092	Opportunism	.517	Self-efficacy	.892
Openness	.223	Proactivity	.374	Autonomy	.569
Extraversion	.472	Creativity	.544	Innovativeness	.738
Neuroticism	− .413	Vision	.573	Internal locus of control	.567
Conscientiousness	.412	META Total	.657	Achievement motivation	.803
				Optimism	.696
				Stress tolerance	.581
				Risk-taking	.788
				BEPE total	.908

Finally, we looked at whether there were statistically significant differences between people who worked for themselves and people who worked for others. The self-employed group (*M*_*θ*_ = 1.20) demonstrated higher mean entrepreneurial personality estimated by CAT than the non-self-employed (*M*_*θ*_ = 1.12), but there were no statistically significant differences (*t* = − 1.508; *p* = .132), and the effect size was small (*d* = 0.13).

## Discussion and conclusions

Interest in the study of entrepreneurial personality has grown considerably in recent years (Brandstätter, 2011; Chandra, 2018; Zhao et al., 2010), as has the creation of measuring instruments to evaluate it. However, to date, these evaluations have not made use of the psychometric advances offered by IRT models, specifically the application of CAT, and this is the objective of our study. A bank of 120 items to measure entrepreneurial personality was developed to be used in a future CAT application, as the two simulations carried out in this study demonstrate. The item bank exhibited good psychometric properties; we confirmed its essentially unidimensional structure and its fit to the Samejima Graded Response Model. The *θ* scores estimated by the CAT were very accurate and correlated very strongly (*r*_*θ*1–*θ*2_ = .948) with the participants’ scores in the full bank of 120 items. This indicates appropriate calibration of the set of items, which is essential for a CAT to work correctly (Olea et al., 1999; Van der Linden & Glas, 2010). In addition, item bank showed no DIF by gender, thus ensuring that gender biased scores are not produced.

The CAT for adults has demonstrated its ability to provide accurate measurements for a wide range of *θ* scores with a small number of items. Through the simulation, we were able to predict what may be expected from the CAT and that this improvement in evaluation efficiency comes without significantly losing accuracy (Barnard, 2018). In most cases, an accurate evaluation by CAT (*M* = 0.157) was achieved with a mean presentation of 16 items.

In terms of evidence of validity, the CAT demonstrates strong correlations with general personality traits such as the Big Five, which makes sense as both approaches (specific vs general traits) predict entrepreneurial success, although predictive capacity is greater using specific traits (Leutner et al., 2014). In addition, there was strong correlation with the META (*r* = .657), one of the most used instruments nowadays for measuring entrepreneurial personality (Almeida et al., 2014), which gives evidence of external validity. Finally, the CAT correlated very strongly (*r* = .908) with the classical version of the BEPE (Cuesta et al., 2018), validating the functioning of the computerized adaptive version. The CAT has various potential fields of application along these lines and is a resource for any type of organization interested in supporting people with high entrepreneurial personalities or in reevaluating people following specialized training in this field. In this regard, the recruitment field can benefit from the use of CAT, as it can provide rapid, online, mass evaluations and is therefore cheaper.

The main limitation was that, despite seeing a tendency of self-employed people to exhibit more entrepreneurial personality (*θ*), the sample of this group was limited (13%), which does not allow us to draw conclusions about the self-employed and non-self-employed or analyze a posteriori whether the CAT correctly discriminates between these two groups, being aware that being self-employed does not necessarily imply having an entrepreneurial personality (Hurst & Pugsley, 2011). In addition, there is no clarification of the type of entrepreneurs that may be found (Hsieh & Wu, 2019). Along these lines, there are other variables that may be considered such as entrepreneurial intent (Hu, Wang, Zhang, & Bin, 2018; Molino, Dolce, Cortese, & Ghislieri, 2018; Newman et al., 2019), dissatisfaction with the current job (Sousa, Araújo, Lua, & Gomes, 2019), and emotional regulation (Castellano, Muñoz-Navarro, Toledo, Spontón, & Medrano, 2019). It is well known that using self-reports leads to many limitations such as acquiescence bias and social desirability bias (Navarro-González, Lorenzo-Seva, & Vigil-Colet, 2016). However, alternatives such as the Implicit Association Test (IAT) have not been shown to be adequate or reliable when evaluating personality traits (Martínez-Loredo, Cuesta, Lozano, Pedrosa, & Muñiz, 2018).

It would be essential in future projects or lines of research to apply the instrument to a subsample of participants in order to check the functioning of the CAT and thus check the results obtained against the simulations (Pedrosa, 2015). In addition, follow-up and re-evaluation of the participants at different time points would make it possible to perform longitudinal studies and observe what leads to business success long term. In addition, as the item bank is constructed from a model with eight facets of entrepreneurial personality (Rauch & Frese, 2007; Suárez-Álvarez & Pedrosa, 2016), an algorithm could be created in the CAT functioning that obliges the use of a determined number of items from each facet, creating a profile of entrepreneurial personality in an adaptive computerized manner. Finally, it would be interesting to differentiate between workers in public and private companies, as well as in different sectors (banking, education, construction, health, human resources), to study the possible differences in responses to the item bank between these groups.

In summary, the present study highlights five important points. First, a computerized adaptive test was developed from a bank of 120 items for the evaluation of entrepreneurial personality. Second, the structure of the item bank was essentially unidimensional and the items were calibrated via the Samejima Graded Response Model. Third, the CAT used a mean of 16 items to evaluate people’s entrepreneurial personality with high accuracy. Fourth, the accuracy of the CAT, evaluated via the information function, was very high for a wide range of scores. Fifth, evidence of predictive validity was produced, with strong correlations between the CAT scores and scores from BEPE and META tests which also evaluate entrepreneurial personality. In short, the CAT for evaluating entrepreneurial personality exhibits good psychometric properties and is an alternative in this field of psychological evaluation for adults.

## Data Availability

The datasets used and/or analyzed during the current study are available from the corresponding author on reasonable request.
